# Infected Urachal Cyst Mimicking Urachal Carcinoma: A Rare Cause of Lower Abdominal Tenderness

**DOI:** 10.5334/jbsr.3029

**Published:** 2023-02-09

**Authors:** Sami Marzouki, Bert Geerts, Jesse Marrannes

**Affiliations:** 1 AZ Sint-Jan Brugge, BE; 2 Ghent University, BE

**Keywords:** Urachal remnants, Urachal cyst

## Abstract

**Teaching Point:** Infected urachal cysts are a rare cause of abdominal complaints and can be accurately diagnosed by abdominal ultrasound and computed tomography (CT).

## Case History

A 57-year-old woman was referred to the emergency department due to abdominal complaints with elevated body temperature and inflammatory blood markers. She complained about obstipation and lower abdominal discomfort since two weeks. Vital parameters, including body temperature were normal. Physical examination showed suprapubic tenderness. Blood analysis showed elevated white blood cell count (13.8 × 10^9^/L) and CRP (167 mg/dL). An abdominal ultrasound revealed a thick-walled heterogeneous cystic mass surrounded by fat stranding anteriorly in the midline on top of the bladder. The bladder dome was thickened, and the lesion was focally invading the left rectus abdominis muscle (Figure 1). Contrast-enhanced abdominal computed tomography (CT) confirmed these findings and showed a continuity between the mass and the median umbilical ligament (Figure 2). The diagnosis of an infected urachal cyst was suggested with suspicion of malignant degeneration based on the focal abdominal wall invasion (dotted arrows). Ultrasound-guided drainage of the mass was performed with evacuation of 70cc purulent fluid. Bacterial culture was positive for S. Anginosus and cytology showed no malignant cells. The patient was successfully treated with antibiotics. Follow-up CT after 2.5 months showed a small residual lesion on top of the bladder dome and disappearance of the extension to the abdominal wall, therefore making the diagnosis of a malignant urachal remnant less probable (Figure 3). The mass was excised, and pathologic examination confirmed the diagnosis of a urachal cyst without malignancy.

**Figure:**
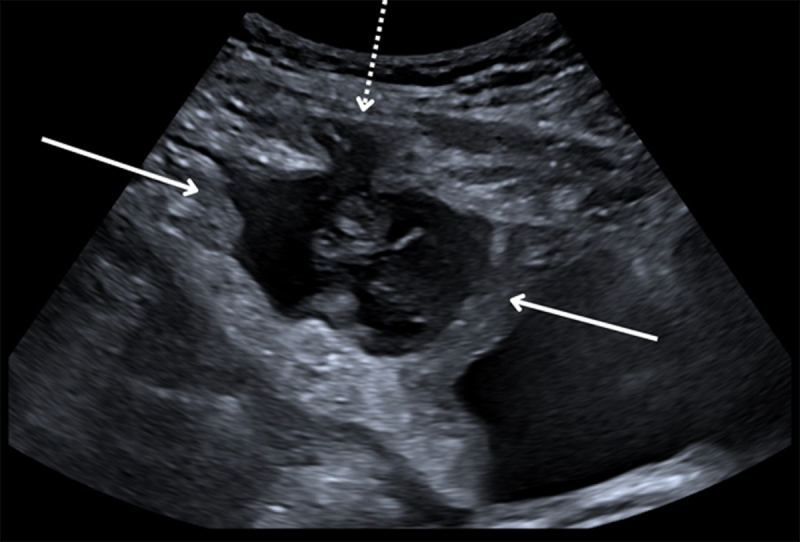
Figure 1

**Figure:**
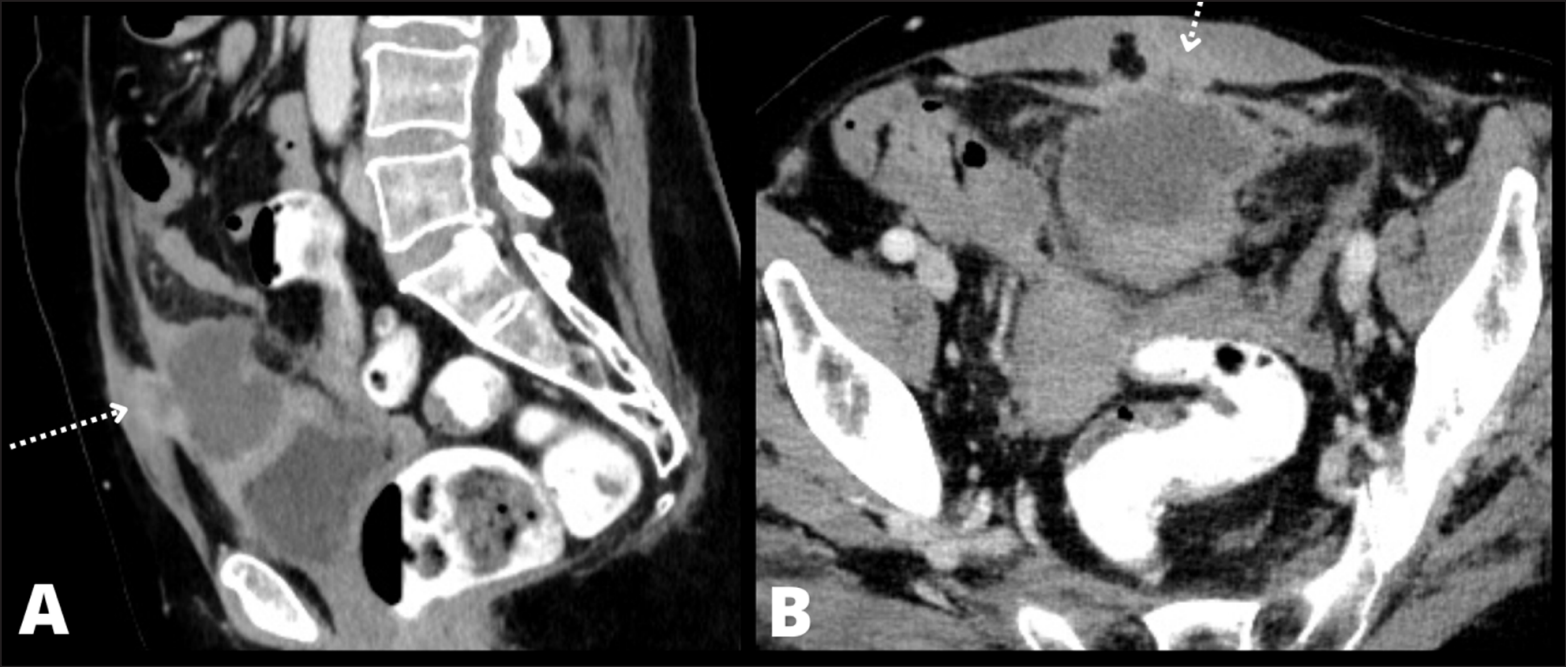
Figure 2

**Figure:**
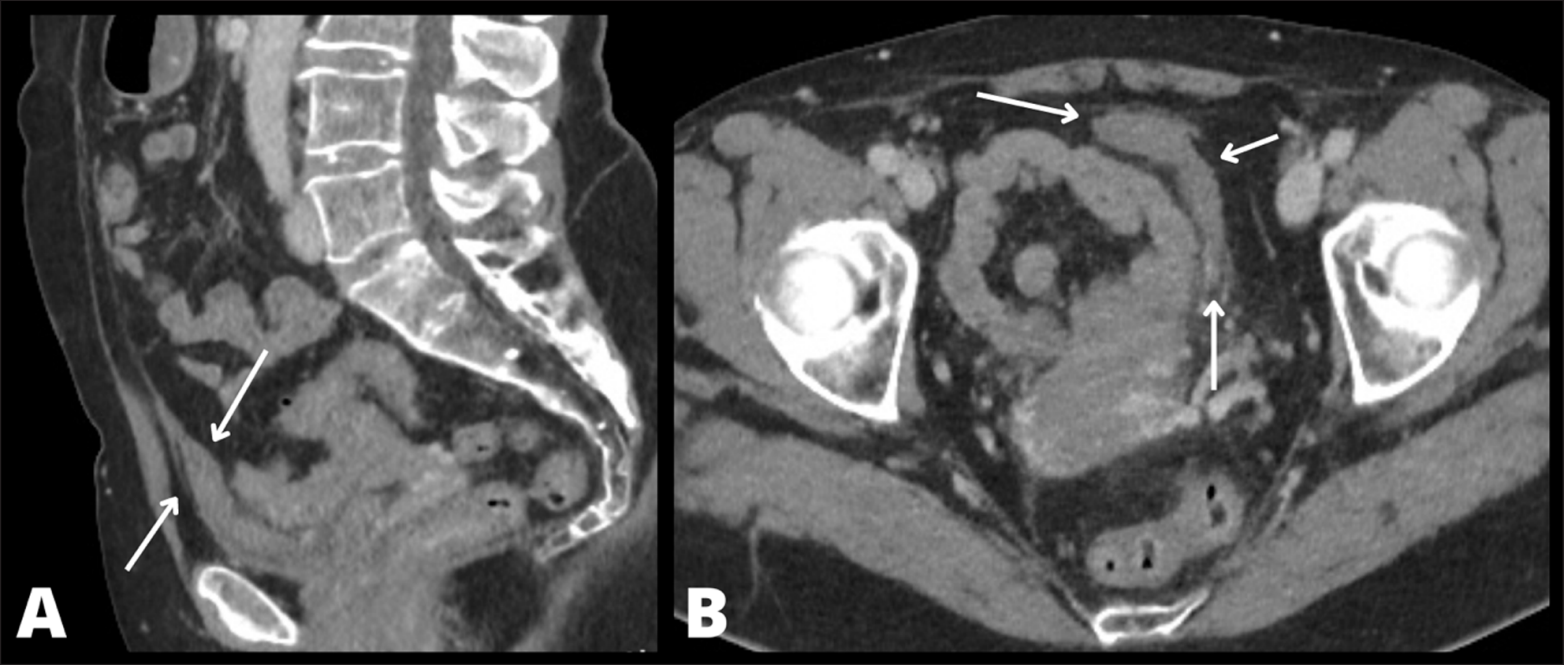
Figure 3

## Comment

The urachus is a ductal embryological remnant, originating from the involution of the allantois and cloaca and extending between the bladder dome and the umbilicus. Urachal anomalies result from failure of the developing urachus to completely obliterate its lumen. Four types of urachal anomalies have been described with a reported incidence of 1/5000 in adults, with the urachal cyst being the most common one. Most anomalies are detected incidentally and more often with the increased use of cross-sectional imaging, in particular CT and magnetic resonance imaging (MRI). The primary imaging modality for urachal cysts is ultrasound revealing a cystic mass in the midline anteriorly between the bladder dome and umbilicus. CT or MRI may be subsequently employed to help reach a definite diagnosis. Infection represents the most common complication of urachal anomalies and may present with nonspecific symptoms. Imaging findings that should prompt consideration of infection in the setting of an acute clinical presentation include the presence of a urachal remnant with complex echogenicity at ultrasound and heterogeneous attenuation of the content and contrast enhancement of the thick wall on CT. As in this case, it can be challenging to distinguish an infected urachal remnant from a malignant urachal neoplasm. Treatment consists of antibiotic therapy followed by complete excision to mitigate the 30% risk of infection recurrence and potential malignant degeneration [1].
